# Scaffold-Hopping Strategies in Aurone Optimization: A Comprehensive Review of Synthetic Procedures and Biological Activities of Nitrogen and Sulfur Analogues

**DOI:** 10.3390/molecules29122813

**Published:** 2024-06-13

**Authors:** Gabriele La Monica, Federica Alamia, Alessia Bono, Antonino Lauria, Annamaria Martorana

**Affiliations:** Department of Biological, Chemical and Pharmaceutical Sciences and Technologies (STEBICEF), University of Palermo, Viale delle Scienze, Ed. 17, I-90128 Palermo, Italy; gabriele.lamonica01@unipa.it (G.L.M.); federica.alamia01@unipa.it (F.A.); alessia.bono01@unipa.it (A.B.); antonino.lauria@unipa.it (A.L.)

**Keywords:** aurones, scaffold hopping, biological activities, organic synthesis, azaaurones, thioaurones, indol-3-one, imidazo [1,2-a]pyridine-3-one, benzothiophenone

## Abstract

Aurones, particular polyphenolic compounds belonging to the class of minor flavonoids and overlooked for a long time, have gained significative attention in medicinal chemistry in recent years. Indeed, considering their unique and outstanding biological properties, they stand out as an intriguing reservoir of new potential lead compounds in the drug discovery context. Nevertheless, several physicochemical, pharmacokinetic, and pharmacodynamic (P3) issues hinder their progression in more advanced phases of the drug discovery pipeline, making lead optimization campaigns necessary. In this context, scaffold hopping has proven to be a valuable approach in the optimization of natural products. This review provides a comprehensive and updated picture of the scaffold-hopping approaches directed at the optimization of natural and synthetic aurones. In the literature analysis, a particular focus is given to nitrogen and sulfur analogues. For each class presented, general synthetic procedures are summarized, highlighting the key advantages and potential issues. Furthermore, the biological activities of the most representative scaffold-hopped compounds are presented, emphasizing the improvements achieved and the potential for further optimization compared to the aurone class.

## 1. Introduction

Natural compounds have always represented an invaluable source of new chemical entities with original and unexplored structures and activities [1,2]. In this context, the flavonoid family is undoubtedly one of the most interesting and studied classes of polyphenol compounds with countless biological properties [3,4].

Aurones, or benzalcoumaranones, represent a class of natural compounds categorized into the group of minor flavonoids. They serve as secondary metabolites and side-products in the biosynthetic pathways of many plant species. Functionally, they contribute to the vibrant and bright color of leaves and flowers as well as the pigmentation of wood, bark, and seedlings, earning them the moniker “golden flavonoids” [5,6,7].

**Figure 1:**
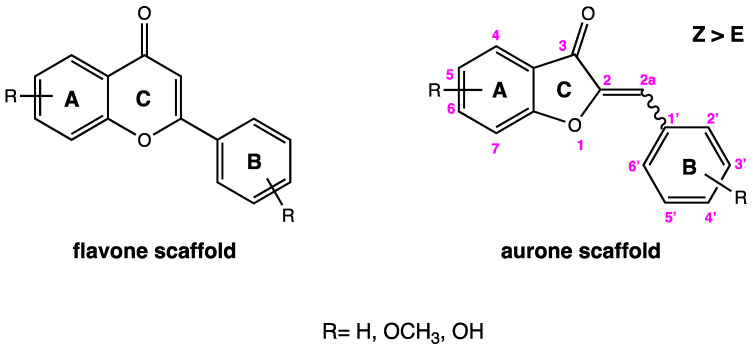
General structure of aurones and their higher structural homolog, flavones, with the three rings A–C and enumeration of atoms evidenced.

As depicted in Figure 1, structurally, aurones are 2-benzylidenebenzofuran-3(2H)-ones, i.e., isomerides and the lower structural counterparts of flavones. They consist of a three-ring system with a central benzofuranone hetero-bicyclic core decorated with a phenyl group on C-2. Unlike flavones, the aromatic side portion is linked through a carbon–carbon exocyclic double bond (2-arylidene portion). Consequently, aurones exist in two different stereoisomeric forms, Z and E. Experimental observations suggest that the Z-stereoisomer is usually thermodynamically preferred in both natural and synthetic derivatives [5,8,9]. In addition, like other polyphenols, the aromatic rings A and B are often polyhydroxylated/methoxylated [5,8].

Due to their scarce presence in nature, aurones have often been overlooked by researchers working in the medicinal chemistry field compared to other flavonoids [10].

However, recent studies have highlighted the intriguing and unique functional properties of aurones. They have demonstrated the ability to selectively modulate biological pathways and specific targets involved in various physiological and pathological processes, including cancer, inflammation, oxidative stress, neurological disorders, and microbial infections [6,11,12]. Consequently, aurones are emerging as privileged scaffolds in the search for new lead compounds, as evidenced by the increasing number of review papers published in recent decades [5,6,7,8,10,11,12,13].

Nevertheless, several physicochemical, pharmacokinetic, and dynamic (P3 features) challenges, common to other natural polyphenols, hinder progress towards advanced stages of drug development. These challenges include limited solubility and cellular permeability, suboptimal bioavailability, and limited metabolic stability due to the ease of oxidation of the polyphenolic framework, as well as a multifunctional profile resulting from promiscuous interactions with numerous off-targets [5]. In this regard, significant efforts have been made to develop synthetic routes to produce semisynthetic/synthetic analogues aimed at conducting extensive structure–activity relationship (SAR) studies and addressing the aforementioned critical aspects of natural aurones [6].

Another promising approach in optimizing natural compounds involves scaffold hopping, which entails substituting the central scaffold of a lead compound with a bioisosteric core to discover more potent compounds with improved P3 features [14,15,16,17,18]. In this context, to the best of our knowledge, there is a dearth of the literature reviewing whether such optimization strategies positively impact aurone compounds.

**Figure 2:**
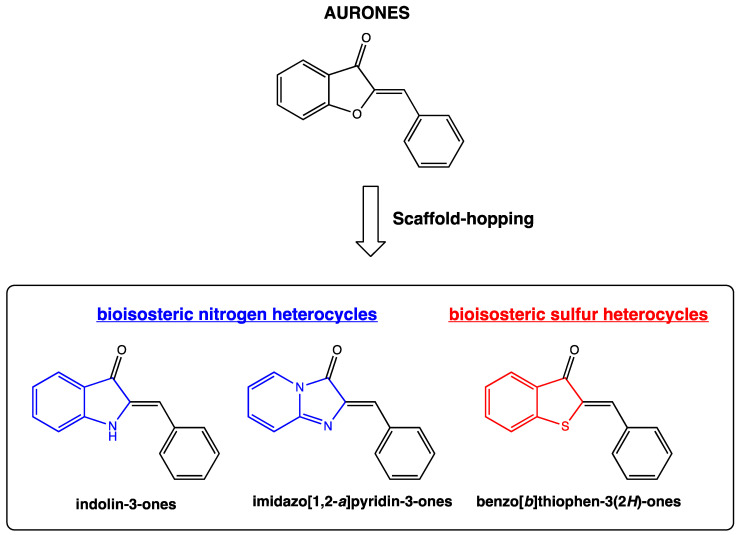
General chemical structure of natural aurones and of the corresponding nitrogen (indolin-3-ones, imidazo[1,2-a]pyridine-3-ones) and sulfur heterocyclic (benzo[b]thiophen-3-ones) analogues reviewed in this overview. The nitrogen scaffolds are evidenced in blue, whereas the sulfur one in red.

This study aims to provide a critical and updated perspective on the impact of scaffold-hopping approaches in this field. In detail, through an in-depth literature analysis, we identified O-to-N/-S substitution as the most common bioisosteric replacement in the medicinal chemistry of natural compounds, including aurones. Figure 2 illustrates the structure of this review paper: the scaffold-hopped aurone derivatives herein evaluated are classified into nitrogen (indolin-3-ones and imidazo[1,2-a]pyridine-3-ones, i.e., aza-/diazaurones) or sulfur (benzothiophenones, i.e., thioaurones) derivatives and discussed in different subsections. In particular, a special focus is given both to the synthetic approaches, highlighting the advantages and limitations of each synthetic method to afford the title scaffolds, and the biological activities, highlighting the differences, especially compared to natural and semisynthetic aurones.

## 2. Bioisosteric Scaffold-Hopped Analogues of Natural Aurones

### 2.1. Indolin-3-One Derivatives (Azaaurones)

#### 2.1.1. Synthetic Procedures: Scaffold Formation

Considering the importance of nitrogen heterocycles in the design of new bioactive small molecules, the simple O-to-N substitution is one of the most studied bioisosteric replacements performed on the aurone scaffold, allowing for the formation of the azaaurone class (or hemiindigos).

**Scheme 1:**
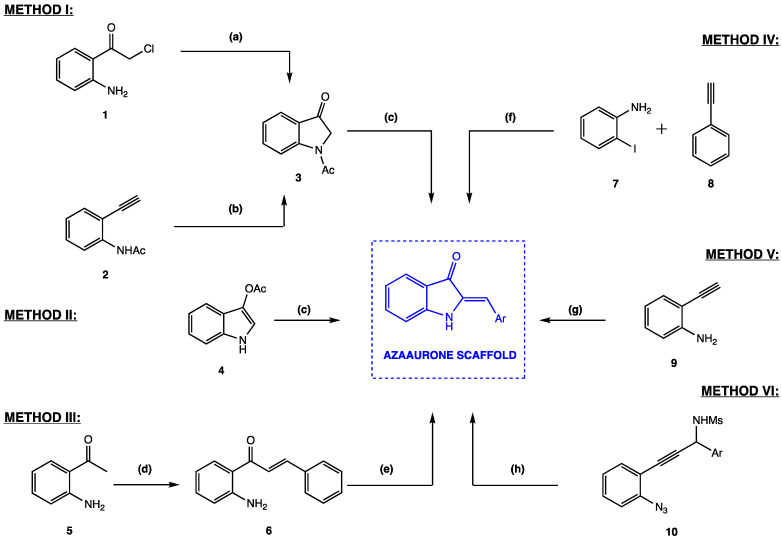
Main synthetic multi-/one-step procedures to afford the (Z)-azaaurone scaffold. Reagents and conditions: (**a**) Ac_2_O, NaH; (**b**) [Au] catalyst; (**c**) base (NaOH, KOH, or aliphatic amine) and aromatic aldehydes; (**d**) aromatic aldehyde, 20% NaOH; (**e**) acetic acid, Amberlyst-15; (**f**) [Pd] catalyst, CO or CO_2_, Et_3_N; (**g**) aromatic aldehyde, 8-methylquinoline-N-oxide, HNTf_2_, [AuCl(JohnPhos)]/AgNTf_2_; (**h**) tBuXPhosAuNTf_2_.

Given their countless applications both in medicinal chemistry and in other scientific fields (materials science, biology, sensing, and optoelectronics), intensive efforts have been made over the last two decades to develop increasingly powerful and convenient methods for the synthesis and derivatization of this interesting scaffold, as described in Scheme 1 (for simplicity, each substituent has been omitted). The azaaurone scaffold can be prepared by traditional synthetic procedures (Methods I–III) or by one-pot procedures (Methods IV–VI), as described in detail in the following sections.

Methods I and II represent the oldest and classical procedures to synthetize the title core and involve the preliminary synthesis of the indolin-3-one/indole building blocks **3** and **4**.

Intermediate **3** can be synthetized by two consecutive steps of N-acetylation and base-catalyzed cyclization of ortho-acylated aniline **1** [19,20,21] or, more rapidly, by a gold-catalyzed (BrettPhosAuNTf_2_ with 8-isopropylquinoline N-oxide) intermolecular oxidation of o-ethynylaniline **2** [22].

On the other hand, 1H-indol-3-yl-acetate **4**, according to traditional methods, is synthetized via a multiple synthetic pathway from isatin/indole derivatives or even purchased from commercial sources [23,24].

Knoevenagel-aldol condensation of **3** and **4** with aromatic aldehydes in the presence of organic or inorganic bases (e.g., piperidine, Et_3_N, NaOH, KOH) allows for selectively obtaining the desired (Z)-azaaurone scaffold [19,20,21,23,24].

Alternatively, in the multi-step Method III, the aldol condensation of 2-aminoacetophenone **5** with aromatic aldehyde provides the amino-chalcone intermediate **6**, which is treated with acetic acid in the presence of a cation-exchange catalyst (Amberlyst-15) to afford the azaaurone scaffold by a selective 5-exo cyclic condensation [25,26].

One-pot Methods IV–VI, employing organocatalyzed cross-coupling reactions, were recently devised and improved with the goal of lowering the number of steps and enhancing the yields.

For instance, in Method IV, 2-iodoaniline **7** and phenylacetylene **8** undergo intramolecular cyclization after a Sonogashira reaction. For this synthetic strategy, to prevent the formation of side-products (such as simple Sonogashira and/or 6-membered ring quinolone compounds), accurate tuning of the reaction parameters (equivalents, temperature, gas pressure, etc.) is required; a temperature of 80 °C, a P_CO_ of 5 bar, and the use of a homogeneous palladium phosphine catalyst, such as Pd(PPh_3_)_4_ (i.e., tetrakis(triphenylphosphine)palladium), allow for selectively affording the Z-azaaurone scaffold [27,28].

Recently, Xiong and co-workers suggested a one-pot approach where the CO intermediate is released in situ from CO_2_ under fluoride-free conditions in the presence of phenylsilane. This protocol showed good functional group tolerance (e.g., methyl, methoxy, halogens, nitrile) and moderate–high yields (average 50–60%) while avoiding the use of CO and providing a safer, milder, and more affordable procedure [29].

Alternatively, Methods V and VI involve a one-pot gold-catalyzed protocol. In the former, the reaction between O-alkynylaniline **9** and aromatic aldehyde affords the title azaaurone scaffold through a protection-group-free double oxidation strategy in the presence of activated phosphane gold complexes as a catalyst (JohnPhosAuCl/AgNTf_2_) and 8-methylquinoline N-oxide as an oxidizing agent [30].

More recently, the novel gold-catalyzed Method VI was described in the literature [31]. This approach entails the nucleophile-controlled trapping of a gold carbene intermediate by water. In detail, the β-sulfonamido-α-imino gold carbene, generated by the gold-catalyzed (tBuXPhosAuNTf_2_) 5-endo-dig cyclization of the N-(2-azidophenyl-ynyl)methanesulfonamide **10**, is captured by water through a concerted intramolecular SN_2_’ reaction to give the final azaaurone core [31].

An overview of all the previously discussed synthetic approaches to afford the azaaurone core is provided in Table 1, highlighting the key aspects, the advantages, and drawbacks of each protocol. A thorough examination reveals that the traditional procedures I-III continue to be the most sought-after and optimized methods for synthesizing this class of compounds, showing many advantages: low cost of the starting materials, even with a wide range of substituents; the use of classical and standard organic chemistry; and no need for expensive catalysts or uncommon laboratory equipment. However, the several synthetic steps required for the synthesis of intermediates **3**, **4**, and **6**, which also involve many protection–deprotection reactions, have a significant impact to the overall yields and reaction time to afford the final products.

On the other hand, the recently proposed protocols IV–VI give the opportunity of a one-pot coordinated synthesis by exploiting a finer transition organometallic catalysis. Such an approach undoubtedly has a positive impact on the overall yield and reaction times. Nevertheless, these protocols still present several issues that limit their extensive use in organic synthesis, especially in medicinal chemistry research, such as expensive or not easily accessible starting materials and catalysts (palladium and gold); the need for unusual laboratory equipment; and the handling of compressed and flammable gases, such as CO.

#### 2.1.2. Synthetic Procedures: Scaffold Elaboration

By exploiting the high versatility of these compounds, other interesting synthetic methods for modifying the azaaurone scaffold have been reported in the literature, providing interesting building blocks for the identification of new biologically active derivatives and further alternatives for SAR exploration.

**Scheme 2:**
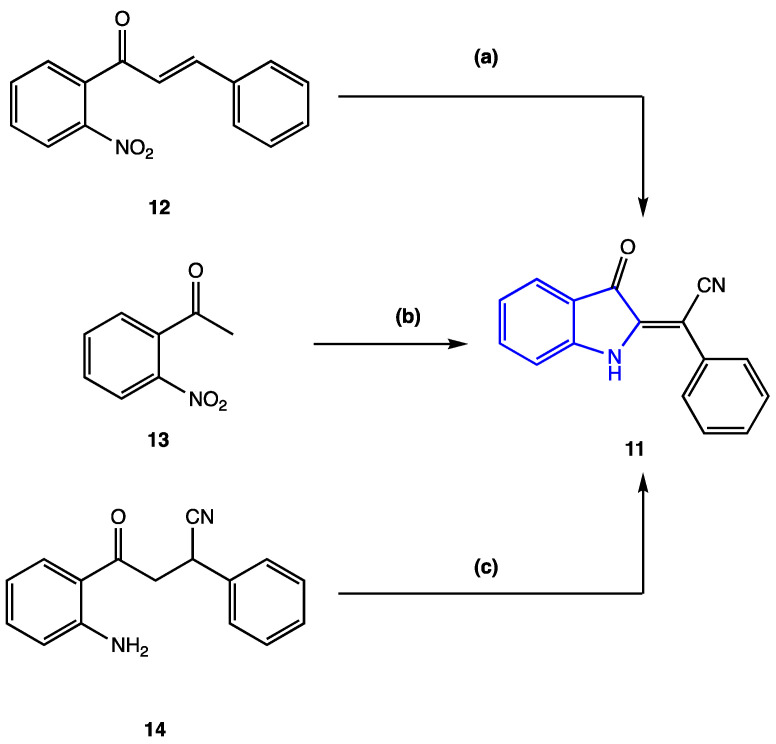
Synthetic protocols proposed by Aksenov et al. to afford the nitrile-substituted azaaurone scaffold of type **11**. Reagents and conditions: (**a**) KCN followed by AcOH; (**b**) aromatic aldehydes, KCN, and then AcOH; (**c**) KOH, DMSO (oxidizing agent).

For example, Aksenov and co-workers have extensively studied protocols to obtain nitrile-substituted azaaurone **11** in a one-pot reaction, as shown in Scheme 2 [32,33,34]. The first method consists of a one-pot cascade transformation/cyclization triggered by the conjugate addition of a cyanide anion (KCN) on ortho-nitrochalcone **12** via a Baeyer–Drewson-like pathway [32]. In a more streamlined synthetic approach, the preparation of a chalcone intermediate and the CN-mediated cyclization is afforded in a single pot from ortho-nitroacetophenone **13** and benzaldehyde (concerted aldol condensation and conjugate addition of cyanide) [33]. The most recent efforts led to a more general and efficient protocol consisting of a one-pot oxidative cyclization of ortho-aminochalcones (e.g., intermediate **14**) assisted by DMSO as an oxidizing reagent [34].

**Scheme 3:**
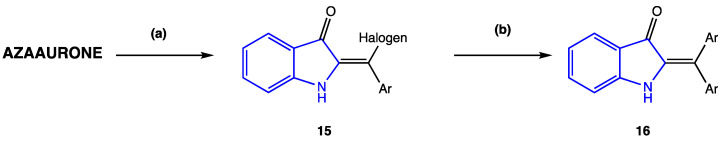
Synthetic method for diarylation of azaaurone compounds. Reagents and conditions: (**a**) N-chlorosuccinimide, MeOH; (**b**) boronic acid, CsF or CsCO_3_, and [Pd^+II^] catalyst.

Another recently reported interesting example of the high versatility of the azaaurone scaffold is the diarylation of the exocyclic double bond, as shown in Scheme 3. In detail, the starting scaffold undergoes to preliminary chemo-selective halogenation through NCS (**15**) followed by a Suzuki–Miyaura cross-coupling reaction with aryl boronic acid to give diarylated azaaurone **16** [35].

#### 2.1.3. Biological Activities

The bioisosteric substitution of the aurone core with the nitrogen-azaaurone heterocycle has been widely explored by medicinal chemists in the last decades and has led to small molecules with promising biological properties. This strategy of lead structure optimization is attracting ever-growing interest, especially in the design and development of new, more effective antimalarial, antimycobacterial, antiviral, and anticancer agents, as described in the following paragraphs.

As a general consideration, most of the biologically active scaffold-hopped compounds herein described were synthetized through traditional procedures (all in all, via Method I described in Section 2.1.1). This witnessed the better suitability and accessibility of such approaches, especially in the drug discovery field, where vast SAR explorations in terms of substituents are necessary and required.

**Figure 3:**
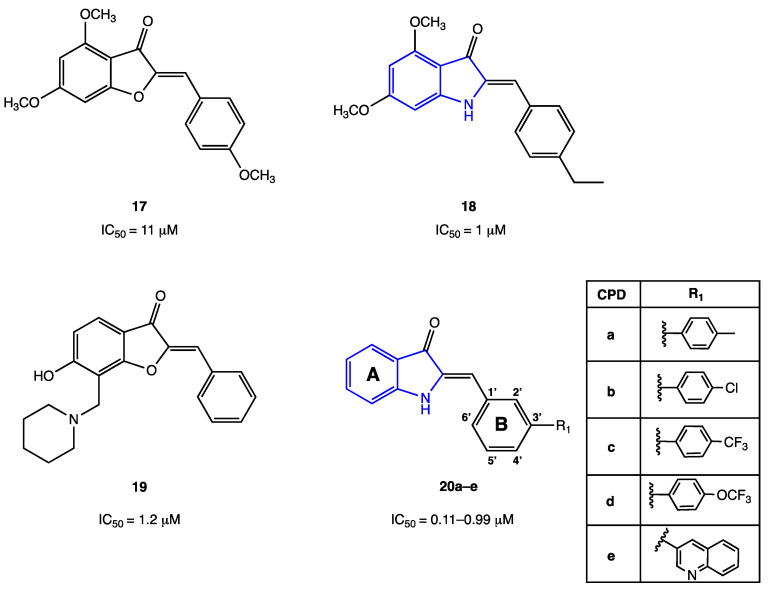
Aurone and azaaurone analogues with potent antimalarial activity against *Plasmodium falciparum*, with the IC_50_ values highlighted. The ring A and B of the central scaffold are evidenced.

In the field of malaria research, several interesting scaffold-hopping approaches to the well-known antiplasmodial aurone core have been pursued, leading to aza-analogues with improved biological activity (Figure 3). For example, Souard and co-workers conducted an extensive SAR study on both aurone and azaaurone series to compare the antimalarial potentials of both scaffolds. In particular, the aza-analogue **18** resulted the most promising one, with better activity in comparison with the best compound from aurone series **17**. In particular, it exhibited a 10-fold improved antiplasmodial activity against various chloroquine-resistant *Plasmodium falciparum* strains, with an IC_50_ of 1 μM, in contrast with the aurone **17**, which exhibited an IC_50_ of 11 μM. Moreover, it showed also a synergistic effect with chloroquine in the FcM29 strain, which was resistant to the approved drug, as well as no signs of acute toxicity after treatment in in vivo studies in mice. The low solubility and bioavailability of compound **18** limited its antimalarial activity in vivo, which was negligible compared to chloroquine [36]. Nevertheless, this first study demonstrated that the endocyclic O-to-N substitution in the aurone series could be bioisosteric, leading to more potent antimalarial agents, and this was the starting point for further studies.

Indeed, in the same field, Carrasco and co-workers also contributed to the understanding of the structural requirements and elucidated the antiplasmodial mechanism of action of this class of compounds, further corroborating the enormous potential of the scaffold-hopping approach in the lead optimization of antimalarial aurones. In a preliminary study, the authors identified synthetic aurone **19** for its IC_50_ in the low micromolar range (1.2 μM) against the chloroquine-resistant *Plasmodium falciparum* W2 strain [37]. Aiming at enhancing the activity of this series, a comprehensive SAR analysis and lead optimization process was performed: compounds **20a–e**, coming from a scaffold-hopping lead optimization process, were the most interesting. With an aromatic moiety at the C3’ position of ring B, they showed more potent antiplasmodial activity against the intraerythrocytic chloroquine-resistant *P. falciparum* W2 strain (IC_50_ in the range of 0.11–0.99 μM) at sub-micromolar concentrations and no cytotoxicity against human embryonic kidney (HEK) 293T cells. Compound **20d** was also tested in an in vitro model for the liver stage of infection in a human hepatoma cell line (Huh7), showing a sub-micromolar EC_50_ value (0.53 μM). This finding suggests it could be a starting point for the development of new and more effective agents with a dual antiplasmodial activity in both hepatic and erythrocytic stages [38].

In addition to the SAR analysis mentioned above, many theoretical computational studies (DFT-based QSAR, HQSAR, 3D-QSAR, COMFA–COMSIA, molecular docking) have been performed to clarify the structural requirements for the antimalarial activity of the azaaurone compounds **18** and **20**. The overall considerations can be summarized as follows: (a) the bioisosteric O-to-NH substitution led to compounds with a more potent antiplasmodial activity; (b) the free NH group is required for the activity, and the N-substitution, such as the N-acetyl group, demonstrated the lower potency of the compounds; (c) the presence of methoxy groups at the C4 and C6 positions of the central azaaurone scaffold, as well as the introduction of bulky substituents, should be considered in the development of new, more potent derivatives; (d) C3’and C4’ substitutions (ring B) with both bulky substituted aromatic (even heterocyclic) or alkyl groups were found in the most active derivatives and seem to be essential for the whole class [36,38,39,40,41,42].

**Figure 4:**
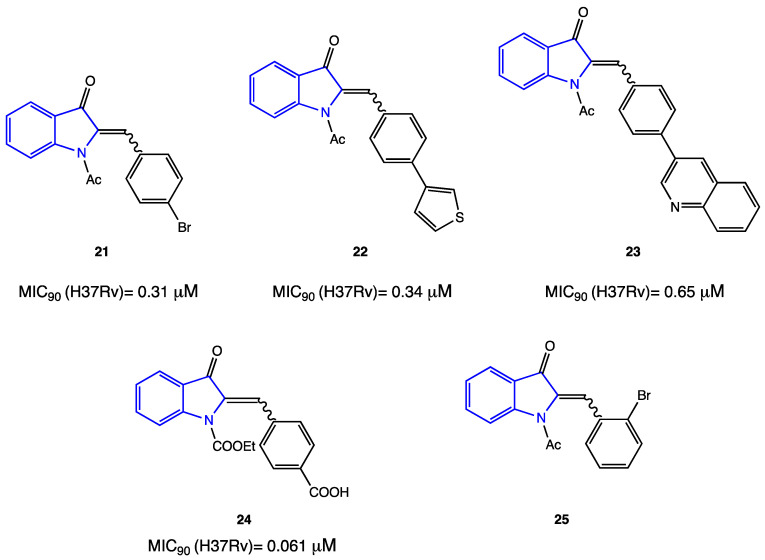
Chemical structure of several azaaurones with anti-*Mycobacterium tuberculosis* activity.

It is noteworthy that Campaniço and co-workers repurposed N-acetylazaaurone compounds, previously identified as weak antimalarials against *Mycobacterium tuberculosis*, to enhance the narrow spectrum of the antitubercular (anti-Mtb) activity of the aurone scaffold. In detail, in a preliminary study, they identified the N-acetyl-azaaurones **21**–**23** (Figure 4) as the most interesting compounds, which are much more potent than their corresponding oxygen counterparts as well as deacetylated azaaurones [43]. Interestingly, as depicted in Figure 4, each compound of this series was isolated in its mixture of E-/Z-stereoisomers due to the presence of a bulky substituent on the endocyclic nitrogen, which probably had a bit of destabilizing effect on the usually more stable Z-stereoisomer.

Indeed, they exhibited stronger activity against the *M. tuberculosis* H37Rv strain, with an MIC_90_ in the sub-micromolar range (0.31–0.65 μM), and they also had a remarkable potency against multidrug-resistant (MDR-TB) and extensively drug-resistant (XDR-TB) strains (MIC in the range of 9.15–15.2 μM). On the other hand, the aurones tested were weakly active, with MIC_90_ values > 20 μM, the maximum concentration tested [43].

However, in vitro metabolic studies showed the lower microsomal stability of the scaffold-hopped analogues due to the NADPH-mediated N-deacetylation. To address this problem, further optimizations of the lead compounds were carried out, focusing on different and less-susceptible N-derivatizations, and in this paragraph, we briefly report the most significant ones. In particular, N-carbamoylation resulted in azaaurone compounds with improved microsomal stability, high kinetic solubility, and improved antimycobacterial activity (in the sub-micromolar range), such as compound **24** (Figure 4) (MIC_90_ H37Rv of 0.061 μM, kinetic solubility of 200 μM, and a CC_50_ > 50 μM against normal cells) [20].

To gain insight into the specific mechanism of action and the in vivo efficacy of this class of compounds, interesting studies were carried out by Yang and co-workers, who conducted an extensive screening of a wide library of aurones and their corresponding nitrogen/sulfur heterocycles for their capability of inhibiting the growth of *Mycobacterium tuberculosis*. In particular, the N-acetylated azaaurone compound **25** (AA2A) (Figure 4), with a 2’-bromo-substituted benzylidene side moiety, showed an 85.95% inhibition of Mtb growth at 100 μM and an MIC of 25 μM. In addition, a potent bacteriostatic effect in an Mtb-infected human macrophage THP-1 cell line along with a significant reduction in the bacterial load in vivo in BALB/c mice was detected. Furthermore, thanks to further biochemical investigations, the inhibition of chorismate synthase, a crucial enzyme of the shikimate pathway involved in the production of key primary metabolites, was identified as the probable mechanism of action for the whole class (IC_50_ of 24 μM) [44,45].

In the examples described above, it emerged that the scaffold-hopping strategy applied to afford the azaaurone compounds in Figure 4 had a dramatic effect on the antitubercular activity; on the one hand, the aurone compounds showed a weak biological activity, and on the other hand, the optimized aza derivatives proved to be an interesting chemical platform to be investigated in the identification of new more potent agents to be used against this infectious disease. In particular, from a general structural analysis (Figure 4), the higher lipophilia of the N-substituted derivatives (N-acetyl or N-carbamoyl groups) in comparison to the corresponding NH and O ones could be responsible for an increased crossing of the mycobacterium, leading to a better antitubercular activity. In addition, in most of the cases analyzed, the substitution at the C4’ of the benzylidene compound with halogens, carboxy, and even heterocycles had a positive effect on the activity.

**Figure 5:**
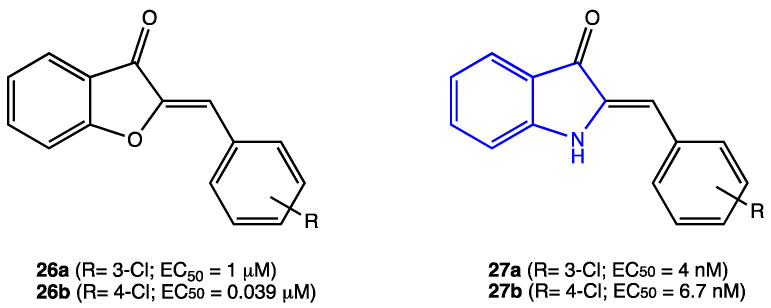
Chemical structure of antiviral azaaurones **27a,b**, and the corresponding aurones **26a,b**; the biological activity (EC_50_, decrease in cytopathic effect) against influenza A/H1N1pdm09 is evidenced.

The method of scaffold hopping to azaaurone appeared to be suitable for developing compounds with potent antiviral activity. For example, aurones **26a,b** were identified for their promising inhibitory activity on neuroamidase NA, a crucial enzyme involved in the replication cycle of influenza A/H1N1pdm09 (a pandemic influenza strain with high virulence). Aiming at discovering new small molecules with improved antiviral activity, Malbari and co-workers performed a bioisosteric O-to-NH substitution. Among all the tested compounds, the chloro-substituted azaaurones **27a,b** (Figure 5) showed the following improved antiviral properties, especially compared to the corresponding aurones **26a,b** and the standard antiviral compounds oseltamivir and quercetin: a better inhibition of the cytopathic effect with increased antiviral and cytoprotective properties (EC_50_ values of 4.0 and 6.7 nM, for **27a** and **27b**; 1.0 and 0.039 μM for **26a** and **26b**; and 12.7 nM and 0.56 μM for oseltamivir and quercetin); a higher selectivity index, i.e., no appreciable toxicity against living host cells at the concentration required for the antiviral effect; and an improved NA-inhibitory activity, with an IC_50_ in the sub-micromolar range (0.52 μM and 3.46 μM, respectively). Furthermore, from both kinetic and in silico studies of induced fit docking, these compounds demonstrated the ability to tightly bind the allosteric 430-cavity, thus acting, as with quercetin, as non-competitive NA inhibitors [25]; such a mechanism of action could also give the possibility of exerting antiviral effects in mutated NA isoforms.

**Figure 6:**
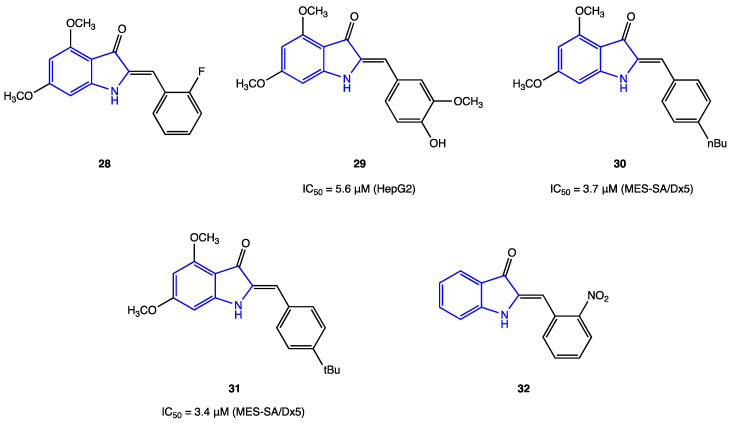
Chemical structure of azaaurone derivatives with promising anticancer activity against aggressive and multidrug-resistant cancer cells.

Several azaaurones, especially with multiple methoxy substitutions, showed remarkable anticancer potentials against various cancer types well known for multidrug resistance, invasiveness, and aggressiveness (Figure 6). Indeed, azaaurone compound **28** (Figure 6) exhibited an interesting antiproliferative activity against bladder cancer cell lines DAG-1 and RT112, with a 50% reduction in cell viability at a concentration of 10 μM, as well as the capability of inducing apoptosis and inhibiting the FGFR3 pathway by a downregulating p-STAT5 and p-Pyk2 expression [19].

Further interesting preliminary antiproliferative data were obtained for compound **29** (Figure 6), retaining the 4,6-dimethoxy-2,3-dihydro-1H-indol-3-one moiety. The scaffold-hopping from oxygen to nitrogen heterocycles significantly increased the antiproliferative effect against hepatocarcinoma cancer cells HepG2 (IC_50_ of 5.6 and 8.4 μM for **29** and the corresponding aurone, respectively) without damaging normal human hepatic cells (WRL-68) [46].

In this context, the dimethoxy derivatives **30** and **31** (Figure 6) were selected from an in vitro screening of more than 100 flavonoid analogues for their potent anticancer effects against multidrug-resistant cancer cells. When tested against human uterine sarcoma cell lines (parental MES-SA and the P-glycoprotein-overexpressing and multidrug-resistant counterpart MES-SA/Dx5), derivatives **30** and **31** strongly and selectively inhibited the proliferation of the MDR-cell line (for **30**, IC_50_ of 17.8 μM and 3.7 μM against MES-SA and MES-SA/Dx5, respectively; for **31**, IC_50_ of 11.9 μM and 3.4 μM against MES-SA and MES-SA/Dx5, respectively), better than well-known flavonoids (e.g., genistein, apigenin) [47].

Interestingly, 2-(2-nitrobenzylidene)indolin-3-one (JHY-A007-50) **32** (Figure 6) was discovered in a biochemical luciferase assay for its capability of inhibiting the expression of the transmembrane prostate androgen-induced protein TMEPAI (IC_50_ of 3.5 μM), a key oncogene that is overexpressed and abnormally activated in several aggressive cancer types, such as lung, breast, colon, and renal neoplasms. Moreover, compound **32** showed a potent cellular activity in several cancer cell lines, with capability of reducing the levels of both TMEPAI mRNA and protein in HeLa, MCF-7, and MGC-803 cells, as well as the arrest of the cell cycle in the G1 phase (downregulation of Cyclin E and CDK2) with a consequent antiproliferative effect [48].

### 2.2. Imidazo[1,2-a]pyridin-3-one Derivatives

#### 2.2.1. Synthetic Procedures

**Scheme 4:**
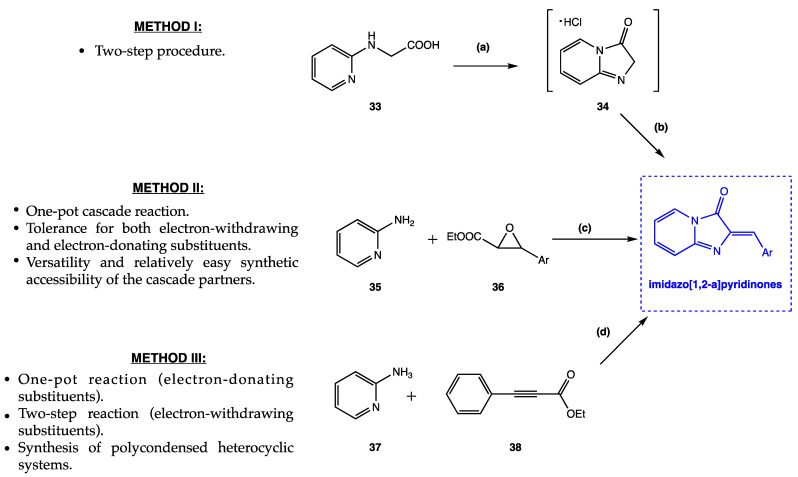
Main synthetic procedures to isolate the imidazo[1,2-a]pyridin-3-one scaffold, a diaza analogue of natural aurone. Reagents and conditions: (**a**) PCl_3_; (**b**) ArCHO, base; (**c**) polyphosphoric acid (PPA); (**d**) PCy_3_. In the right part, an overview of the most interesting features of each synthetic process is given.

The imidazo[1,2-a]pyridin-3-one scaffold is one of the most interesting diaza bioisosteric analogues of naturally occurring aurone. Due to its peculiar structure, there are few and relatively new synthetic methods described in the literature to obtain this heterocycle (Scheme 4).

In Method I, the imidazo[1,2-a]pyridinone derivative is prepared by a multi-step procedure involving the intramolecular cyclization of the N-2-pyridinyl-glycine derivative **33** with PCl_3_ to afford the bicyclic intermediate **34** (Ia). The aldol condensation of **34** with various aromatic aldehydes by using pyridine/NEt_3_ as both bases and solvents allows for the isolation of the diazaaurone core (Ib) (Scheme 4) [49,50].

In recent decades, the interesting biological activities of aurone and its derivatives have led the scientific community to develop more efficient, simpler, and versatile synthetic procedures.

In this context, Method II consists of the one-pot cascade reaction between 2-aminopyridine **35** and aryl 2,3-epoxyethylesters **36** to afford the desired (Z)-2-arylideneimidazo [1,2-a]pyridinones (Scheme 4). Heating to 110 °C under neat conditions and the use of polyphosphoric acid (PPA) as a catalyst and multifunctional activator resulted in optimal reaction conditions to produce the title compounds in considerable yields (60–70%). The use of polar aprotic solvents (DMSO, DMF, and MeCN) and Brönsted acids other than PPA (p-TsOH, TfOH, AcOH, MsOH, and HClO_4_) provided a complex mixture of by-products, with a drastic reduction in the desired product yields. The high tolerance to both electron-withdrawing and electron-donating groups in the starting materials is certainly an interesting advantage of this preparation process, allowing for the introduction of a variety of functionalities such as halogens (chloro, bromo) and nitro and alkoxy groups, which can be easily manipulated in case of further structural optimization processes. From a mechanistic point of view, this method exploits the tendency of the oxirane ring to act as a bielectrophile. In detail, the mechanism consists of a unique process: cleavage of the epoxydic C–O bond, the formation of an α-enamine ester via a PPA-mediated aziridine intermediate, and intramolecular transamidation with only water and ethanol as byproducts [51,52].

Another interesting one-pot method for the synthesis of the arylidene-imidazo[1,2-a]pyridin-3-one scaffold (Method III, Scheme 4) consists of an organocatalyzed umpolung addition of 2-aminopyridine **37** to arylpropiolate **38**, followed by a concerted ester–lactam exchange reaction. Among all the catalyst, tricyclohexylphosphine (PCy_3_) proved to be the most effective, especially in the presence of electron-donating groups on the starting materials. However, in the presence of electron-withdrawing groups, the intermediate enamine ester did not undergo direct cyclization, and the heating with PPA allow for obtaining the cyclized products, even with various hetero-polycyclic-2-amidines [53].

In the right part of Scheme 4, the key aspects of the methods analyzed above for the synthesis of the imidazo[1,2-a]pyridin-3-one scaffold are listed. In detail, Methods II and III seemed to be the most effective and versatile procedures due to their one-step strategy, high tolerance to various substituents, and the possibility of carrying out the reactions under solvent-free conditions. Method I, on the other hand, requires two synthetic steps, but no expensive catalysts, and rather classical/well-known organic chemistry reactions.

#### 2.2.2. Biological Activities

Several imidazo[1,2-a]pyridin-3-one derivatives have been described in the literature as scaffold-hopped analogues of natural aurones, exhibiting improved biological properties (Figure 5). In contrast with azaaurones, in the case of diaza analogues, the one-pot Methods II–III furnished an optimal and ideal synthetic toolbox to be applied for scaffold-hopping campaigns. Indeed, the high tolerance for both electron-withdrawing and electron-donating substituents along with the high versatility and relatively easy synthetic accessibility of the cascade partners make these procedures ideal for medicinal chemists interested in in-depth SAR explorations.

**Figure 7:**
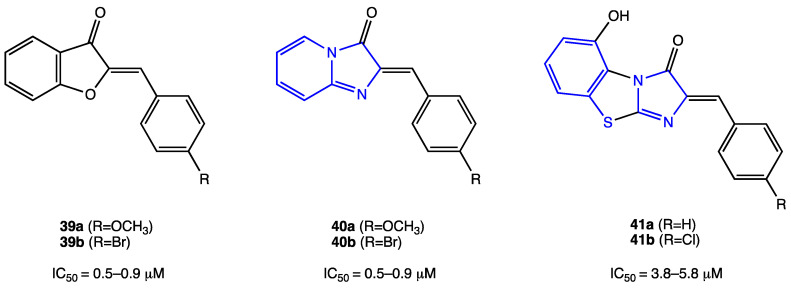
Chemical structure of imidazo[1,2-a]pyridin-3-one derivatives endowed with anticancer properties, with the range of antiproliferative activity (expressed as IC_50_) against multiple cancer cell lines evidenced.

In the field of anticancer activity, the imidazo[1,2-a]pyridin-3-one compounds **40a**,**b**, with a methoxy and a bromo substituent on the respective aryl moiety (Figure 7), showed interesting antiproliferative and TopoII-inhibitory activities, better than the corresponding aurones **39a**,**b** and the reference agent etoposide. In particular, compounds **40a,b** demonstrated a lower relative hTopo-mediated decatenation of kinetoplast DNA, with a selective poisoning of the TopoIIα isoform, as also confirmed in ex vivo experiments. In addition, in in vitro cellular assays, they displayed multifold higher potent anticancer activity against both renal (HEK-293T) and breast cancer (MCF-7, MDA-MB-231, and MDA-MB-468) cell lines (for **40a** and **40b**, IC_50_ in the range 45–74 nM; for the corresponding aurones **39a**,**b**, IC_50_ in the range 0.5–0.9 μM), proapoptotic effects, the inhibition of tubulin polymerization, and the disruption of microtubule dynamics with consequent cell cycle blockage in the G2/M phase [52].

Through an iterative scaffold-hopping strategy applied on this first series of diaza aurone analogues, several (Z)-2-benzylidenebenzo[d]imidazo [2,1–b]thiazol-3-ones, such as **41a**,**b**, were designed by introducing a thiazole ring within the imidazo-pyridinone bicyclic system (Figure 7). Biological assays evidenced the stronger antiproliferative activity of the title compounds compared to the reference compound etoposide, with a high selectivity towards cancer cell lines compared to normal cells (IC_50_ 3.8–5.8 μM against MCF-7, MDA-MB-231, HEK-293T, and H-357); moreover, they were also able to block the cell cycle in the S phase, to downregulate the expression of anti-apoptotic proteins (BCL-XL, BAX), and to significantly inhibit Topoisomerase [53]. A comprehensive structural analysis highlighted that for both bicyclic and tricyclic compounds, the substitution with methoxy or halogens in ring B is essential to achieve the desired biological activities.

These studies demonstrated that even scaffold-hopping to diaza heterocycles could be pursued to optimize the biological activity of aurones.

### 2.3. Thioaurone Derivatives

#### 2.3.1. Synthetic Procedures

Sulfur heterocycles also represent important building blocks in the design of new bioactive small molecules; for this reason, O-to-S bioisosteric substitution has been pursued, even if less frequently than the N one, in the lead optimization of aurone analogues, allowing for affording the (Z)-thioaurone class (or hemithioindigos).

**Scheme 5:**
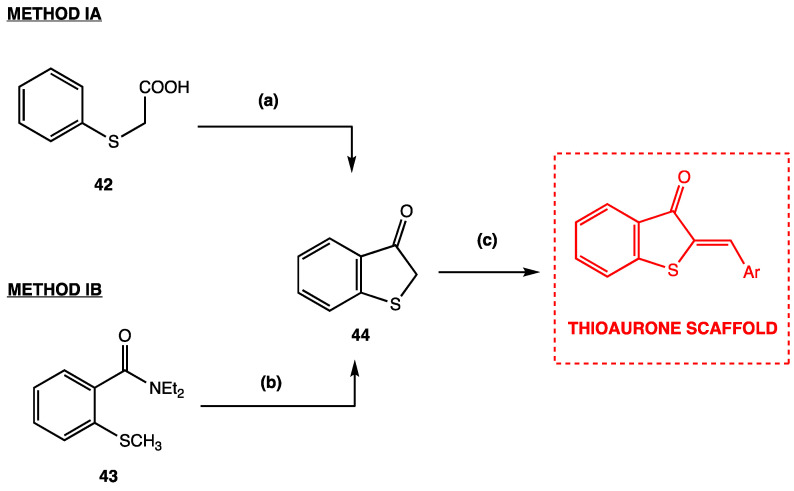
Main synthetic multi-step procedures to afford the thioaurone scaffold from 1-benzothiophen-3(2H)-one intermediate. Reagents and conditions: (**a**) (1) SOCl_2_, (2) AlCl_3_; (**b**) LDA, −78 °C; (**c**) ArCHO, with piperidine/NaOH/basic aluminum oxide (basic conditions) or CH_3_COONa under acidic catalyst (acid conditions).

**Scheme 6:**
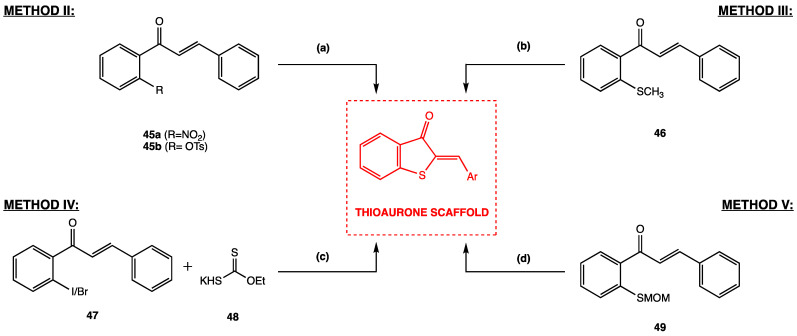
Main synthetic procedures to afford the thioaurone scaffold starting from chalcone analogues as reaction intermediates. Reagents and conditions: (**a**) S_8_, tertiary amine (e.g., Et_3_N or N-methylpiperidine), DMSO; (**b**) I_2_, DMSO; (**c**) CuI, I_2_; (**d**) NBS, pyridine.

**Scheme 7:**
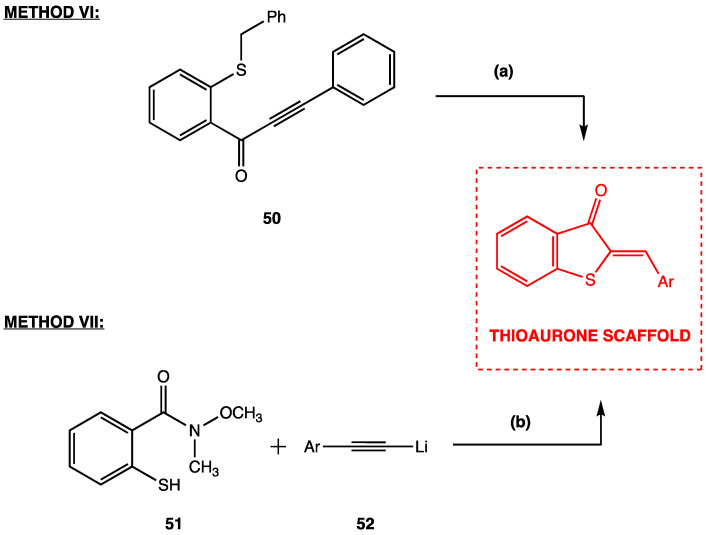
Main synthetic one-pot procedures to afford the thioaurone scaffold. Reagents and conditions: (**a**) formic acid; (**b**) LDA.

However, considering their significant application not only in the medicinal chemistry field but also in the field of molecular photo-switches (e.g., application in photo-pharmacology, diagnosis, imaging, and in the production of dyes, cosmetics production, and chemical sensors), several synthetic procedures have been reported in the literature in the last two decades, as described in Scheme 5, Scheme 6 and Scheme 7 (the substituents were omitted for an easier general view). In detail, the synthetic methods for the preparation of the thioaurone scaffold can be divided into three different classes: multi-step procedures in which 1-benzothiophen-3(2H)-one is the key intermediate (Methods IA,B, Scheme 5); multi-step procedures involving a chalcone analogue as an intermediate (Methods II–V, Scheme 6); and one-pot procedures involving an alkyne intermediate (Methods VI–VII, Scheme 7), as described in detail in the following paragraphs.

The classical method to obtain the thioaurone scaffold involves the preliminary synthesis of 1-benzothiophen-3(2H)-one **44** (Methods I, Scheme 5), which is achieved following two different main protocols described in the literature. According to Method IA, compound **44** can be prepared through the Friedel–Craft acylation of α-phenylthioacetic acid **42** (easily accessible by the alkylation of thiophenol with 2-chloroacetic acid) using SOCl_2_ as a chlorinating reagent and AlCl_3_ as a Lewis acid [54,55]. Method IB, instead, involves a lithium diisopropylamide (LDA)-mediated in situ anionic cyclization of N,N-diethyl-2-(methylthio)benzamide **43**, which is achieved by the directed ortho-metalation of the corresponding benzamide followed by quenching with dimethyl disulfide [56,57,58]. Intermediate **44** undergoes aldol condensation with aromatic aldehydes using piperidine/NaOH/basic aluminum oxide under basic catalyst conditions or acetic acid and sodium acetate under acidic catalysis to give the title thioaurone scaffold [54,55,56,57,58,59,60].

On the other hand, Methods II–V involve a chalcone analogue as the intermediate. This is synthetized through the aldol condensation of an appropriate starting ketone and aldehyde, and it then undergoes cyclization under various conditions (Scheme 6).

Method II considers the direct cyclization of the chalcone intermediate bearing a good leaving group (Csp^2^-NO_2_, Csp^2^-OTs) with elemental sulfur (S_8_) and comprises two different variants. In the first variant, 2′-nitrochalcone **45a** reacts with elemental sulfur in the presence of aliphatic amines (such as N-methylpiperidine, Et_3_N, or DIPEA) and DMSO as additives, which act as activators of S_8_ towards the nucleophilic aromatic substitution. The reaction temperature depends on the substituents; rt conditions work well in the case of chalcones substituted with halogen- or electron-withdrawing substituents (NO_2_ or CF_3_), whereas mild-to-moderate heating (60–100 °C) is required in the case of electron-donating (methoxy, amine, amide) or sterically hindered groups [61,62,63].

In the latter, 2’-tosylchalcone **45b** is subjected to a cascade reaction involving the incorporation of elemental sulfur and subsequent cyclization, favored by both the highly reactive tosyl leaving group and the ortho electron-withdrawing carbonyl. A base catalysis (e.g., Et_3_N), DMSO as a solvent, and mild heating (80 °C) are required to maximize the yields. Even if a wide range of substituents are tolerated at both the aromatic portions of the chalcone intermediate (methoxy, methyl, nitro, and halogens), the major limitation of this method is that cyclization could also occur via the β attack to enone, yielding the six-membered heterocyclic compound (thioflavone) as a major product (60–70%) [64].

Method III, instead, involves a metal-free vinylic carbon-hydrogen bond thiolation of chalcone intermediate **46** (ortho-methylthiophenyl vinyl ketone) catalyzed by iodine (50% mol.). The reaction needs to be conducted in DMSO as a solvent and requires heating above 150 °C. However, with this procedure, the thioflavone scaffold is obtained in a lower yield (7–20%) than with the previous protocol, thus representing a minor subproduct [65].

In Method IV, the orto-halogenated (I or Br) chalcone **47** is cyclized to a thioaurone core through the reaction with xanthate **48**, an odorless sulfur surrogate. In the presence of CuI and I_2_ as catalysts, the chalcone is selectively iodinated in the α position, with the consequent univocal cyclization of the saturated ketone to the thioaurone nucleus; thanks to this peculiar mechanism of action, the cyclization to the six-membered thioflavone is avoided [66].

In very recent times, the mild and straightforward Method V has been proposed. In this protocol, the access to the title scaffold is afforded by means of the NBS-mediated cyclization of the methoxymethyl(MOM)-protected mercapto-chalcone **49** at rt with a high functional group tolerance [67].

On the other hand, two one-pot procedures have been also proposed by Lee, as reported in Scheme 7 (Methods VI–VII). In the first synthetic approach, (Z)-thioaurone can be synthesized by debenzylation followed by an intramolecular 5-exo cyclization of 1-(2-benzylthio)phenyl-3-phenyl-2-propyn-1-one **50**, derived from thiosalicylic acid, using formic acid in THF. The presence of a benzyl group is crucial to selectively direct the cyclization completely towards the 5-exo mode; in the case of an alkyl group (e.g., n-Bu) the 6-endo cyclization, which affords the corresponding thioflavone core, is favored [68].

In Method VII, instead, the one-pot synthesis of the title core is accomplished by the regioselective LDA-catalyzed addition/cyclization of arylethynyllithium **52** on N-methoxy-N-methyl-2-mercaptobenzamide **51** [69]. Both procedures exhibited high tolerance against the most recurrent substituents.

As in the previous paragraphs, in Table 2, a summary of all the synthetic protocols to obtain the thioaurone scaffold is provided, highlighting the potential advantages and drawbacks of each of the described methods. By an in-depth analysis, even in the case of sulfur heterocycles, the multi-step procedures (I–V) are the most pursued, optimized, and explored in terms of substituents. In detail, procedure I, which starts from a 1-benzothiophen-3(2H)-one intermediate, takes the advantages of starting from relatively inexpensive and easy-to-find starting materials as well as a high tolerance for a wide range of substituents explored. However, since this protocol involves a lot of synthetic steps, the overall yields could be negatively affected; in addition, the handling of organosulfur compounds (sulfides and thiols) is discouraged.

Regarding multi-step procedures involving chalcone compounds (II–V), they share several advantages (high functional group tolerance) and disadvantages (multi-step procedures) with the previous method. It should be noted that two protocols (II and IV) do not involve the handling of any organosulfur compound, but instead, they make use of a sulfur surrogate such as elemental sulfur S_8_ or xanthate. However, in some cases (II and III), a lack of regioselectivity has been observed; the 6-endo cyclization of chalcone competes with the 5-exo one, affording an undesirable mixture of the required five-membered thioaurone compound with the corresponding six-membered thioflavone as a side-product, even with non-negligible yields.

Methods VI and VII undoubtedly represent a starting point for the development of more efficient procedures; on the one hand, they take the advantage of a potential one-pot synthesis, but on the other hand, they have not been explored enough, the starting materials appear to be not easily accessible (neither commercially nor synthetically), and they involve the handling of an organolithium intermediate, for which special equipment and expertise are required.

#### 2.3.2. Biological Activities

Due their peculiar structural and electronic features, thioaurone compounds are of interest in many scientific areas, such as photo pharmacology, diagnosis, imaging, and in the production of dyes, cosmetics production, chemical sensors, advanced materials, systems, and devices [70,71,72].

**Figure 8:**
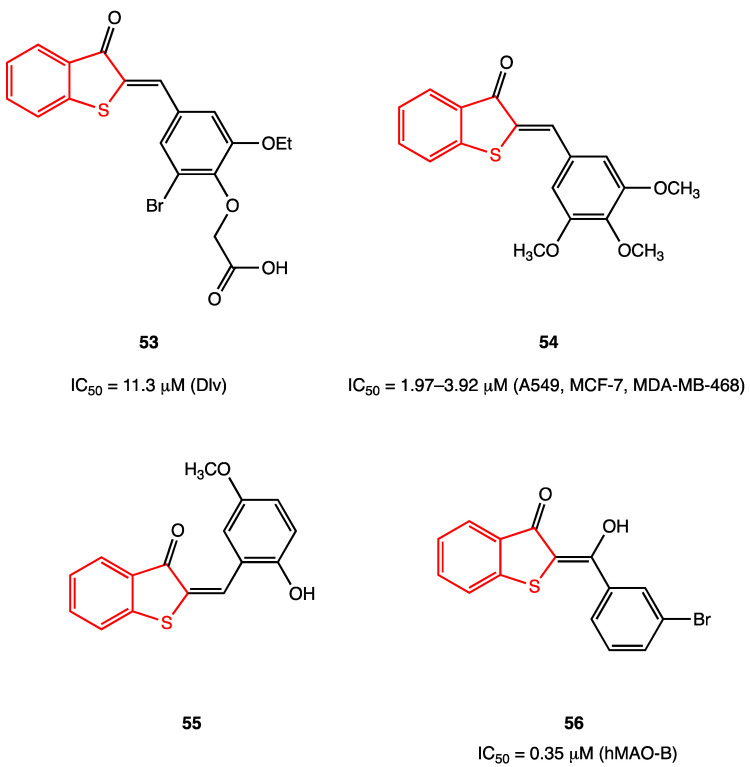
Chemical structure of thioaurone compounds endowed with biological activity.

Regarding its application in a medicinal chemistry context, the thioaurone scaffold has been less explored than the corresponding nitrogen analogue. In this section, several examples of the most interesting and fruitful thioaurones endowed with biological activity (anticancer, anti-inflammatory, antidiabetic, and neuroprotective/antioxidant) are described (Figure 8).

In the anticancer field, numerous thioaurones have shown remarkable antiproliferative activity by selectively inhibiting targets involved in neoplastic processes. For example, compound **55** (BMD4702) was discovered through a structure-based pharmacophore modelling study for its capability to bind and modulate the Dlv protein, an effector of the Wnt/b-catenin signaling pathway with multifaceted functions involved in cell growth and cancer development. Compound **53** was potentially able to bind the PDZ domain of Dlv, with an IC_50_ of 11.3 μM and a k_D_ of 0.186 μM. Docking studies corroborated the in vitro results, confirming the importance of the benzothiophenone scaffold in forming stabilizing H-interactions with crucial amino acids within the PDZ cleft (Arg^325^) as well as hydrophobic contacts [73].

Accordingly, in recent times, Kaur et al. reported a wide series of hemithioindigo derivatives with potent Topoisomerase I/II inhibitory activity as a new template for the design of new more effective and selective anticancer compounds. Among them, **54** (Figure 8) was found to be the most potent antiproliferative derivative, with an IC_50_ in the range of 1.97–3.92 μM against the highly resistant lung cancer A549 and breast cancer MCF-7 and MDA-MB-468 cell lines, better than the references campthotecin (CPT) and etoposide (IC_50_ in the ranges of 7.41–8.92 μM and 6.71–23 μM, respectively), as well as the capability of arresting the cell cycle in the G1/S phase. In addition, in in vitro relaxation DNA studies, compound **54** was also able to inhibit both Topo I and II with a potency comparable to CPT, and this was confirmed also by in silico docking studies, where the benzothiophenone ring proved to be essential in binding the crucial magnesium ion (MG903) within the Topoisomerase catalytic pocket [62].

In the area of anti-inflammatory agents, compound **55** (BAS00795786) emerged from an in silico virtual screening analysis of a database of flavone analogues as a potent inhibitor of 5-lypooxygenase (5-LO), an enzyme involved in the production of pro-inflammatory mediators, showing a remarkable binding affinity in docking, molecular dynamic simulations, and MM-GBSA studies, as well as favorable ADME properties [74].

Several thioaurone analogues also showed interesting neuroprotective activity, better than the corresponding aurone analogues. In this light, Guglielmi and co-workers, aiming at enhancing the human monoamine oxidase B (hMAO-B) inhibitory activity and antioxidant properties of set of aurone derivatives (IC_50_ 11.6–26.3 μM), conducted a scaffold-hopping study in this series to afford the corresponding benzo[b]thiophen-3-ol/thioaurone analogues. Compound **56** proved to be the most interesting of the series, with an IC_50_ against hMAO-B in the sub-micromolar range (0.35 μM) and a high selectivity towards this isoform, in comparison to MAO-A (selectivity index of 180), as also confirmed by molecular docking simulations. In addition, to assess its neuroprotective properties, it was tested in cortex synaptosomes in both basal and LPS-induced inflammatory conditions, showing the capability of reducing the DA/DOPAC ratio as well as LDH activity [75].

## 3. Conclusions and Future Directions

Aurones, due to their scarce presence in nature in contrast with other polyphenolic compounds (indeed, they belong to the class of minor flavonoids), have been often overlooked in the past decades with respect to their possible interest in a medicinal chemistry context. In the last few decades, the interest for this class of compounds has increased due to their outstanding biological properties, especially in the field of antiviral, anticancer, and antimalarial agents [6,11,12]. Despite this, considering their unfavorable P3 properties, an acceptable drug-likeness is still far from being achieved [5]. In this light, the scaffold-hopping method is probably one of the most interesting approaches to be pursued in the lead optimization of natural compounds [14,15,16,17,18].

For this reason, in this review paper, we aimed to provide an overview of the most interesting examples of scaffold-hopping strategies applied in the optimization of aurone compounds, focusing especially on nitrogen (indolin-3-ones and imidazo[1,2-a]pyridine-3-ones) and sulfur (benzothiophenones) heterocyclic analogues.

To give a comprehensive panorama of the topic, in addition to the impact of scaffold-hopping on biological activity, special attention was firstly paid to the analysis of the synthetic procedures reported in the literature to afford the described heterocycles. In the first instance, it emerged that all the references cited were published in the last fifteen years, and this is strictly related to the relatively recent interest in these classes of derivatives. As for the natural oxygen counterparts, the synthetic procedures developed to date allow for affording the Z-analogues as the most abundant/unique stereoisomers, and this is due to their higher thermodynamic stability.

In detail, by comparing the synthetic methods described, the type of chemistry available to date can be classified into three different reaction types, with the only difference in the heteroatom considered: (1) traditional methods consisting of the aldol condensation between a previously synthetized bicyclic central scaffold (**3** or **4** in the case of azaaurones; **34** for diazaaurones; **44** for thioaurones) with aromatic aldehydes; (2) the intramolecular cyclization of a chalcone intermediate (e.g., **6**, **45**–**49**); and (3) one-pot condensation reactions catalyzed by transition metals (such as for intermediates **7**–**10**, **37**, and **38**, **50**–**52**).

From this study, it emerged that the former procedures still represent the most used ones, especially in the medicinal chemistry field. This is witnessed by the synthetic methods pursued to obtain most of the bioactive heterocyclic aurone analogues herein described. This could be ascribed to the easier organic chemistry involved as well as the possibility of more extensive and vast SAR studies, with less limitations in terms of substituents, peculiar reaction conditions, and the need for unusual catalysts. However, several issues were also evidenced; the multi-step synthetic pathway involved frequently led to a drastic reduction in overall yields. Furthermore, especially in those procedures involving chalcone as an intermediate, a lack of regioselectivity could be observed, with the possibility of producing intramolecular cyclization by-products different from the desired ones. On the other hand, despite the potential advantages in terms of time and resources, one-pot procedures still need to be optimized to make them more accessible, sustainable, and cost-effective for the whole scientific community. Thus, a key area of focus for future research should be the development of more streamlined and sustainable synthetic routes (minimization of waste generation), with less use of expensive and rare catalysts, hazardous reagents, and solvents (green chemistry principles). This will allow for expanding the synthetic toolbox to afford the heterocyclic aurone analogues, enabling the rapid and efficient construction of diverse molecular architectures and then the generation of structurally diverse libraries of aza-/thioaurone derivatives for biological screening and structure–activity relationship studies.

In this light, the interest in the development of optimal and effective synthetic procedures to afford aza- and thioaurones has been accompanied by a notable interest in these compounds in medicinal chemistry research, as demonstrated by the examples of scaffold-hopping described in this overview. In particular, from this point of view, the O-to-N substitution resulted in the most fruitful and viable series of compounds with outstanding biological activities and drug-like properties that are much more improved in comparison with their aurone counterparts (see Section 2.1.2 and Section 2.2.2). These compounds have better antimalarial, anticancer, and antiviral activity, better solubility and metabolic stability, and improved interactions with the target proteins. Furthermore, the possibility of N1 derivatization for nitrogen derivatives, which is not feasible for thio analogues, allows for wider SAR exploration (e.g., compounds **21**–**25**), opening up new chemical spaces and biological activities that are scarcely found in aurone derivatives (antimycobacterial activity). In this regard, it can be noted that the presence of bulky substituents at the nitrogen atom have led, surprisingly, to compounds in mixtures of their Z-/E-stereoisomers. This experimental evidence needs to be further explored, and hypothetical different biological activities and potency of the two stereoisomers should be investigated and elucidated.

On the other hand, the biological properties of thioaurone, even if interesting (anticancer, anti-inflammatory, and neuroprotective), proved to be less explored than the nitrogen counterparts; thus, they deserve to be investigated more in the near future.

In conclusion, in this review, we provided a critical analysis of the scaffold-hopping approaches directed towards the lead optimization of aurone compounds. In our opinion, the detailed evaluation of both the synthetic approaches and the biological activities, especially in comparison with the aurone class, could be an important and useful foundation in the development of new more efficient and streamlined synthetic methods and biologically active bioisosteric derivatives of the aurone class. By continuing to explore innovative synthetic methodologies and elucidate the biological activities of the heterocyclic aurone derivatives, researchers can unlock new opportunities for the development of efficacious therapeutics targeting a diverse array of diseases.

## Figures and Tables

**Table 1:** Overview of the most interesting synthetic procedures to afford the azaaurone.

Synthetic Procedure	Key Points	Ref.
I–III	Multi-step procedure (possible drastic reduction in the overall yield). Need for protection–deprotection steps (e.g., acetyl group introduction/removal). Classical organic synthesis reactions and common laboratory equipment. Wide pattern of substitutions both on rings A and B by modification of commercial starting materials (anilines/2-amminoacetophenones and aldehydes). No need for expensive transition metal catalysts.	[19,20,21,22,23,24,25]
IV	One-pot cross-coupling reaction: optimized yields (50–60%). Use of palladium organic catalysts [Pd]/PR_3_. More expensive starting materials (e.g., iodoanilines). Accurate control of the reaction parameters to avoid side-products. Need for highly toxic and flammable pressurized gas (in ex situ CO generation only); a milder protocol with CO in situ generation is preferable. Good tolerance for a wide range of functional groups. Need for special laboratory equipment (e.g., glovebox, autoclave)	[27,28,29]
V–VI	One-pot reactions. Starting products not easily accessible. Need for a strictly inert atmosphere. Use of quite expensive and uncommon gold catalysts. More limited applications and fewer substituents explored.	[30,31]

**Table 2:** Overview of the most interesting synthetic procedure to afford the thioaurone analogues.

Synthetic Procedure	Key Points	Ref.
IA,B	Multi-step procedures: possible drastic reduction in the overall yields. Relatively inexpensive and easy-to-find starting materials. Tolerance for a wide range of substituents. Several steps involve malodorous organosulfur compounds.	[54,55,56,57,58]
II–V	Multi-step procedure to afford the desired chalcone intermediate. No need for organosulfur compounds (e.g., Methods II and IV, with the use of elemental S_8_ or xanthate). Tolerance for a wide range of substituents. Lack of regioselectivity and formation of thioflavone byproducts (in Methods II and III, the 6-endo cyclization competes with the 5-exo one).	[61,62,64,65,66]
VI–VII	One-pot reaction. Starting products not easily accessible. Handling of organolithium compounds. More limited applications and fewer substituents explored.	[68,69]
